# Congenital soft tissue Ewing's sarcoma: A case report of pre- and postnatal magnetic resonance imaging findings

**DOI:** 10.1097/MD.0000000000028587

**Published:** 2022-01-14

**Authors:** Chaoxiang Yang, Wenjun Chen, Penghui Han

**Affiliations:** Department of Radiology, Guangdong Women and Children's Hospital, Guangzhou, China.

**Keywords:** diffusion weighted imaging, magnetic resonance imaging, prenatal, soft tissue Ewing's sarcoma

## Abstract

**Rationale:**

Most congenital soft tissue masses are benign. Ewing's sarcoma (ES) is a highly malignant tumor that commonly occurs in children and adolescents and rarely occurs during the fetal period. Cases of congenital soft tissue ES with magnetic resonance imaging (MRI) findings are scarce. To the best of our knowledge, no previous reports have described the pre- and postnatal MRI findings of ES.

**Patient concerns:**

We present a case of congenital soft tissue ES arising in the body wall, which was examined using MRI during the prenatal and neonatal periods.

**Diagnoses:**

Malignancy was suspected by diffusion-weighted imaging, which demonstrated restricted diffusion within the mass even during the fetal period. ES was confirmed via histopathological examination after birth.

**Interventions:**

The patient initially underwent conservative treatment for suspected hemangioma. Tumorrectomy was undergone after three weeks based on previously dissatisfied therapeutic effects.

**Outcomes:**

The patient died of multiple distant metastases despite undergoing postoperative chemotherapy and metastasectomies.

**Lessons:**

Fetal or neonatal soft tissue ES may be clinically misdiagnosed as a hemangioma. It is important to suspect this through an imaging approach such as diffusion-weighted imaging.

## 1 Introduction

Fetal soft tissue masses are occasionally encountered in clinical practice. Most congenital solid masses such as hemangiomas and teratomas are benign. However, although uncommon, malignant soft tissue masses that develop during the prenatal and neonatal periods have poorer outcomes.

Ewing sarcoma (ES) is a highly malignant tumor that commonly occurs in children and adolescents. However, this rarely occurs during the fetal period. Congenital extraskeletal ES tumors are typically located in the extremities, pelvis, or soft tissues of the trunk. Herein, we report a congenital soft tissue ES on the body wall, which was documented using pre- and postnatal magnetic resonance imaging (MRI).

## 2 Case report

**Figure 1:**
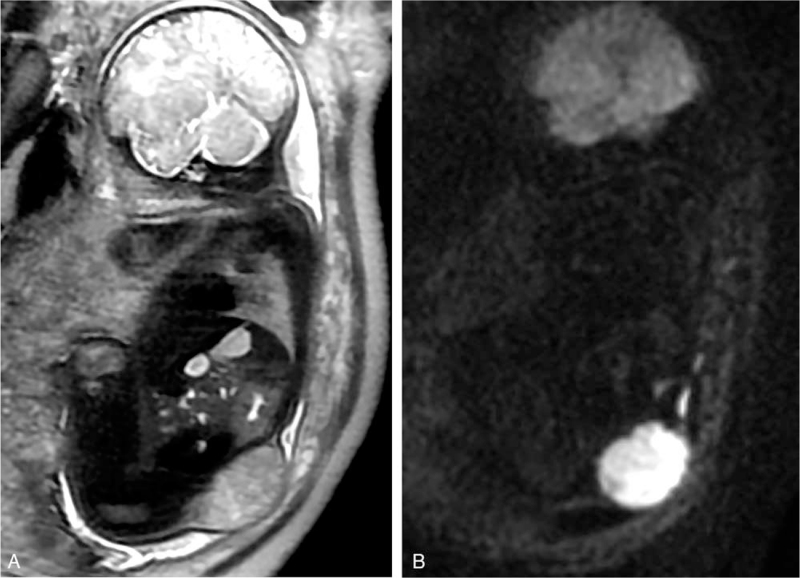
Sagittal T2-weighted imaging at 37 weeks of gestation showed a mixed-signal subcutaneous solid mass located on the left lumbar region (A). On sagittal DWI (b = 800 s/mm^2^), the mass demonstrated significantly high signals (B).

A 29-year-old woman (gravida 2, para 1) was referred and admitted to the hospital at 37 weeks of gestation for ultrasonographic detection of a fetal body wall mass. Her medical history was unremarkable and she was not taking any medications during her pregnancy. Antenatal screening revealed a low risk of trisomies 13, 18, and 21. The maternal viral serological test results were negative. Prenatal ultrasound showed a mixed echogenic mass on the lateral side of the left lower abdominal wall of the fetus, with abundant blood flow signals inside the mass and along its margins. No other abnormalities were observed in the patient. Subsequent prenatal MRI revealed a large subcutaneous solid mass located in the left lower lumbar region (Fig. 1A and B). The mass measured 57 × 63 mm in diameter, with a heterogeneous signal on T2-weighted imaging and an extremely high signal on diffusion-weighted imaging (DWI). No signs of adjacent infiltration or intra-abdominal extension were observed. These features suggested a congenital tumor of the body wall, and malignancy was suspected because of restricted diffusion on DWI. Chromosomal analysis revealed normal karyotype.

**Figure 2:**
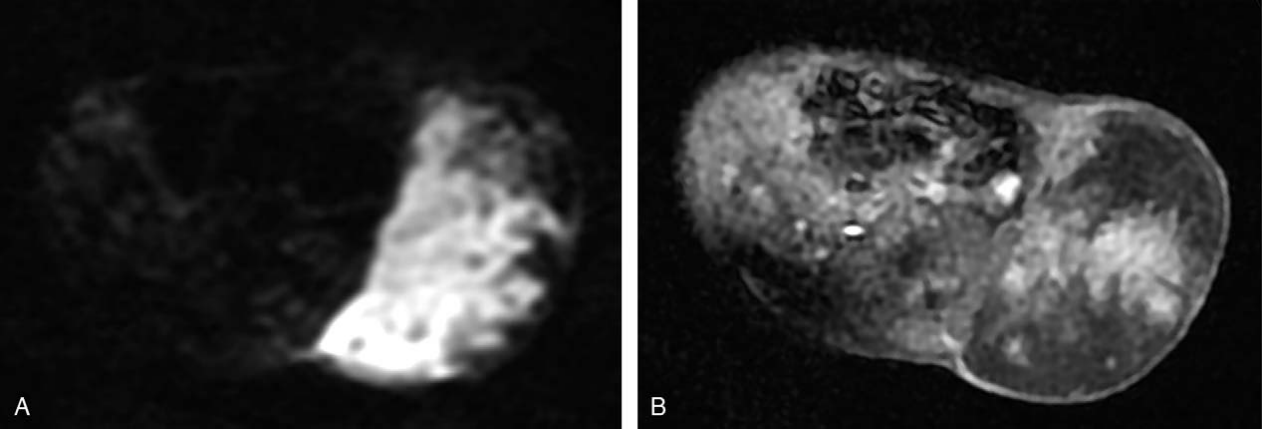
On postnatal axial DWI (b = 800 s/mm^2^), the mass showed marked hyperintensity similar to that observed in prenatal imaging (A). Enhanced axial T1-weighted imaging showed a heterogeneously enhanced mass (B).

**Figure 3:**
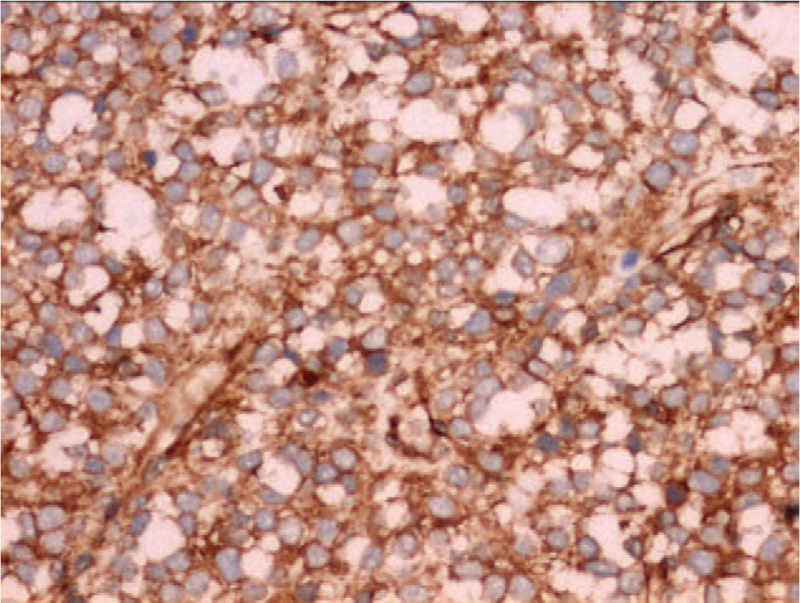
The tumor cells are positive for CD 99 (IHC, × 200).

At 38 weeks and three days of gestation, a live male infant with a birth weight of 2800 g was delivered vaginally. The infant had Apgar scores of 9 and 10 at 1 and 5 min, respectively, and did not require resuscitation. Physical examination revealed a large hyperpigmented soft-tissue mass with normal skin temperature on the left side of the infant's abdomen. MRI done two days after birth revealed a mass measuring 67 × 77 mm in diameter with low T1 and heterogeneous high T2 signal intensity. As observed on prenatal imaging, the mass exhibited marked hyperintensity on DWI (Fig. 2A). Contrast-enhanced T1-weighted imaging with fat saturation further confirmed the heterogeneously enhanced mass (Fig. 2B), suggesting its malignant potential. Initially, the patient underwent conservative treatment because hemangioma was suspected. However, the mass continued to enlarge after the administration of propranolol. Three weeks after birth, the neonate underwent tumorectomy and the mass was completely resected along with some underlying muscles. Gross inspection of the mass revealed a fish-flesh-like cut surface with scattered areas of hemorrhage. Histopathological examination of the mass confirmed the presence of small, round malignant tumor cells with a margin that was clear of the residual tumor cells. The tumor cells were immunohistochemically positive for CD99 (Fig. 3) and Ki67 (95%) and negative for synaptophysin, CD117, neuron-specific enolase, inhibin, and epithelial membrane antigen. The final pathological diagnosis was ES. After a month and a half, distant metastasis was detected in the scalp after undergoing chemotherapy at another hospital. There were no signs of local recurrence, and the metastatic tumor was resected. One month later, multiple metastases involving the brain, lungs, and scalp were detected by physical examination and imaging, following which the infant became emaciated and sick, and eventually died of the disease.

## 3 Discussion

ES belongs to the Ewing sarcoma family of tumors, which include primitive neuroectodermal tumors (PNETs) and Askin tumors.^[1]^ The incidence of ES peaks at 10 to 15 years of age, and most cases occur in the skeletal system. Congenital ES (i.e., ES occurring in fetuses and neonates) is rare, and few cases of soft tissue ES have been reported. Among the 34 cases of congenital ES and PNET documented by Haas et al^[2]^ 18 occurred in the extraskeletal tissues, including the head and neck, body wall, upper limb, abdomen, and retroperitoneum. To date, approximately 20 congenital ES/PNET cases have been reported in soft tissues previously.^[2–5]^ However, few of these cases have documented MRI results. To the best of our knowledge, this is the first reported case of congenital ES with MRI findings from both the pre- and postnatal (neonatal) periods.

Yildiz et al^[3]^ reported a case of fetal ES that presented as a cervical mass containing cystic and solid areas on T2-weighted images. Case reports of postnatal ES with MR images obtained during the neonatal period are scarce.^[6–8]^ In these few reports, soft-tissue ES masses were characterized by heterogeneous and hyperintense enhancement. Several of the masses had necrotic or cystic areas and involved the adjacent tissues. However, DWI was not included in any previous pre- or postnatal ES reports. The DWI technique is a special MRI sequence developed to detect water diffusion in vivo. Currently, it is the only non-invasive method that provides crucial diagnostic information regarding the presence or absence of restricted diffusion. The restricted diffusion is due to the high density of malignant cells and scant cytoplasm, both of which strongly indicate malignancy.^[9]^ Malignant tumors visualized with DWI exhibit highly restricted diffusion as opposed to the free diffusion observed in benign tumors (such as hemangiomas). In the current case, we suspected malignancy based on DWI findings.

The main differential diagnoses include hemangioma, infantile fibrosarcoma, and teratomas. Fetal hemangiomas in the body wall are more common than those in subcutaneous ES. Hemangiomas have low-intermediate or predominantly high signals on T2-weighted images. Masses containing more vessels and a reticulated mixture exhibited a mixed-signal intensity with decreased intensity dots and increased intensity voids. A hemangioma usually exhibits isointense or slightly hypointense signals on DWI owing to unrestricted water diffusion, which is a differential point with ES and other malignant tumors. Congenital fibrosarcoma and extraskeletal ES are similar, in terms of the sites of predilection and imaging findings. Both of these pathologies present with high signal intensity on DWI, and differentiating between the two via prenatal MRI evaluation is difficult. However, fibrosarcoma has a better prognosis than ES, and rarely metastasizes. Fetal subcutaneous teratomas primarily occur in the sacrococcygeal, oral, and maxillofacial regions. Teratomas exhibit infiltrative spread and calcified cystic areas on MRI, which are not typically observed in subcutaneous ES.

Currently, the treatment of choice for congenital ES includes radical surgical resection, chemotherapy, and radiotherapy. Despite these treatments, the disease has a poor prognosis, and most diagnosed newborns do not survive for more than two years.^[10]^ Based on the currently unfavorable prognosis of fetal soft tissue ES, it is crucial to investigate the possibility of prenatally diagnosing this disease. We suggest that MRI, including DWI, should be performed when prenatal ultrasound reveals a solid soft tissue tumor. If an apparent high signal intensity is observed in a fetal mass using DWI, clinicians should suggest a potential malignancy so that appropriate prenatal counseling can be offered to parents.

## 4 Conclusion

Subcutaneous soft tissue ES during the fetal or neonatal period may be misdiagnosed as a hemangioma. MRI techniques, particularly DWI, can play an important role in the differential diagnosis of benign and malignant masses.

## Author contributions

**Resources:** Wenjun Chen, Penghui Han.

**Writing – original draft:** Chaoxiang Yang.

**Writing – review & editing:** Chaoxiang Yang, Wenjun Chen.
